# A loading dose of clofazimine to rapidly achieve steady-state-like concentrations in patients with nontuberculous mycobacterial disease

**DOI:** 10.1093/jac/dkae309

**Published:** 2024-10-08

**Authors:** Ralf Stemkens, Arthur Lemson, Simon E Koele, Elin M Svensson, Lindsey H M te Brake, Reinout van Crevel, Martin J Boeree, Wouter Hoefsloot, Jakko van Ingen, Rob E Aarnoutse

**Affiliations:** Department of Pharmacy, Research Institute for Medical Innovation, Radboud University Medical Center, Nijmegen, The Netherlands; Department of Pulmonary Diseases, Research Institute for Medical Innovation, Radboud University Medical Center, Nijmegen, The Netherlands; Department of Pharmacy, Research Institute for Medical Innovation, Radboud University Medical Center, Nijmegen, The Netherlands; Department of Pharmacy, Research Institute for Medical Innovation, Radboud University Medical Center, Nijmegen, The Netherlands; Department of Pharmacy, Uppsala University, Uppsala, Sweden; Department of Pharmacy, Research Institute for Medical Innovation, Radboud University Medical Center, Nijmegen, The Netherlands; Department of Internal Medicine and Infectious Diseases, Research Institute for Medical Innovation, Radboud University Medical Center, Nijmegen, The Netherlands; Department of Pulmonary Diseases, Research Institute for Medical Innovation, Radboud University Medical Center, Nijmegen, The Netherlands; Department of Pulmonary Diseases, Research Institute for Medical Innovation, Radboud University Medical Center, Nijmegen, The Netherlands; Department of Medical Microbiology, Research Institute for Medical Innovation, Radboud University Medical Center, Nijmegen, The Netherlands; Department of Pharmacy, Research Institute for Medical Innovation, Radboud University Medical Center, Nijmegen, The Netherlands

## Abstract

**Objectives:**

Clofazimine is a promising drug for the treatment of nontuberculous mycobacterial (NTM) diseases. Accumulation of clofazimine to reach steady-state plasma concentrations takes months. A loading dose may reduce the time to steady-state-like concentrations. We evaluated the pharmacokinetics (PK), safety and tolerability of a loading dose regimen in patients with NTM disease.

**Methods:**

Adult participants received a 4-week loading dose regimen of 300 mg clofazimine once daily, followed by a maintenance dose of 100 mg once daily (combined with other antimycobacterial drugs). Blood samples for PK analysis were collected on three occasions. A population PK model for clofazimine was developed and simulations were performed to assess the time to reach steady-state-like (target) concentrations for different dosing regimens.

**Results:**

Twelve participants were included. The geometric mean peak and trough clofazimine concentrations after the 4-week loading phase were 0.87 and 0.50 mg/L, respectively. Adverse events were common, but mostly mild and none led to discontinuation of clofazimine. Our loading dose regimen reduced the predicted median time to target concentrations by 1.5 months compared to no loading dose (3.8 versus 5.3 months). Further time benefit was predicted with a 6-week loading dose regimen (1.4 versus 5.3 months).

**Conclusion:**

A 4-week loading dose regimen of 300 mg once daily reduced the time to target clofazimine concentrations and was safe and well-tolerated. Extending the loading phase to 6 weeks could further decrease the time to target concentrations. Using a loading dose of clofazimine is a feasible strategy to optimize treatment of NTM disease.

**Clinical Trials Registration:**

NCT05294146

## Introduction

Nontuberculous mycobacterial (NTM) diseases are increasingly reported worldwide and the most common clinical manifestation is NTM pulmonary disease (NTM-PD).^1,2^ Antibiotic treatment of NTM diseases requires multidrug regimens, characterized by long treatment durations, frequent occurrence of adverse effects, and poor cure rates.^3,4^

Clofazimine is an old antibiotic that is primarily used in the treatment of leprosy.^5^ In more recent years, there has been an increased interest in the use of clofazimine for the treatment of NTM disease and drug-resistant tuberculosis (DR-TB).^1,6–8^ Clofazimine is highly active in vitro against clinically important NTM species, i.e. *Mycobacterium avium* complex (MAC) and *Mycobacterium abscessus* complex (MAB), and shows synergy with clarithromycin and amikacin.^9,10^ Clofazimine-based regimens have also shown comparable efficacy to first-line rifampicin-based regimens in patients with MAC-PD and it is considered a first-line oral antibiotic for the treatment of MAB-PD.^1,11–13^

While clofazimine has been increasingly recognized as an important drug, studies evaluating exposure–response relationships are scarce and the optimal dose of clofazimine is unknown.^14^ The dose that is commonly used for NTM disease (and DR-TB) is 100 mg once daily, although a range of 100–200 mg daily is recommended for NTM-PD.^1^ In addition, there is experience with higher daily doses of 300 mg daily in the treatment of leprosy and DR-TB.^14–17^ Clofazimine is characterized by complex pharmacokinetics (PK). It is highly protein-bound, very lipophilic, and accumulates particularly in adipose tissue and macrophage-rich organs. This results in a very long elimination half-life of ∼30–70 days. As a result, it takes several months to reach steady-state (‘stable’) concentrations in plasma.^6,18^ This implies that the drug is not contributing fully to the NTM treatment regimen for months. We believe that treatment efficacy could be improved by using a higher dose at the start of treatment, i.e. a loading dose, to faster achieve concentrations similar to those at steady-state (steady-state-like concentrations). The use of loading doses is not uncommon for drugs that, similar to clofazimine, have long elimination half-lives, such as the anti-TB drug bedaquiline.^19^ To date, a loading dose strategy has not been evaluated for clofazimine in the treatment of NTM disease.

We assessed the PK, safety, and tolerability of clofazimine in patients with NTM disease who received a loading dose regimen of 300 mg once daily for 4 weeks followed by a maintenance dose of 100 mg once daily. Furthermore, we aimed to identify the optimal loading dose regimen of clofazimine using a population PK model-based approach.

## Methods

### Study population

Adult patients with pulmonary or extrapulmonary NTM disease, eligible for treatment with clofazimine, were included in this study (see Supplementary Table S1 (available as Supplementary data at *JAC* Online) for in- and exclusion criteria). Written informed consent was obtained from all participants.

### Study design

This explorative, one-arm, open-label, PK study (acronym C-LOAD) was performed at Radboud University Medical Center (Radboudumc), Nijmegen, the Netherlands. Owing to the explorative nature of the study, no sample size calculations were performed. We aimed to enrol 10 participants but allowed for additional enrolments (a maximum of five), in case of early withdrawals. The study was approved by an ethical review board, METC Oost-Nederland, and was conducted under Good Clinical Practice standards. This study is registered with ClinicalTrials.gov under trial number NCT05294146.

**Figure 1.**
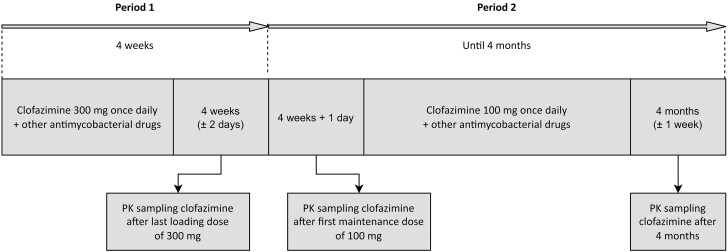
Schematic overview of the study periods and PK sampling occasions. Adherence to treatment and adverse effects were also monitored on PK sampling occasions as well as on additional study visits: day 1, 1 week, 2 months and 3 months (phone call). ECGs were recorded 2–6 hours post-dose of clofazimine on day 1, at 4 weeks and 4 months and, without a specific time window, after 1 week and 2 months.

All participants received a loading dose regimen of clofazimine (Lamprene), 300 mg once daily for 4 weeks (period 1), followed by a maintenance dose of 100 mg once daily until a total study duration of 4 months (period 2; see Figure 1 for a schematic overview). Other antimycobacterial drugs were used as per standard of care, following an international guideline, throughout the study.^1^ With the loading dose regimen, we aimed to reach steady-state-like concentrations of clofazimine in a shorter time period. The loading dose regimen was selected based on explorative simulations with a population PK model for TB patients.^18^ Clofazimine was administered with food, as recommended by the manufacturer and because this improves absorption.^6^ Adherence to clofazimine was evaluated using pill counts (only in period 1), a medication diary and self-assessment by participants.

### PK blood sampling and bioanalysis

Blood samples for assessment of PK parameters of clofazimine were collected on three occasions (Figure 1). Samples were collected after the last loading dose of 300 mg (at 4 weeks (±2 days), with sampling time points at pre-dose and at 2, 4, 6, 8 and 24 hours post-dose. In addition, samples were collected after the first maintenance dose of 100 mg (at four weeks + 1 day), with sampling time points at 2 and 6 hours post-dose, and once more after 4 months (±1 week). Total plasma concentrations of clofazimine were measured using validated liquid chromatography–mass spectrometry methods (details are shown in Table S2).

### Monitoring of safety and tolerability

Participants were monitored throughout the study for evidence of clinical or laboratory-based adverse events (AEs). Severity grading of AEs occurred according to the Common Terminology Criteria for Adverse Events (CTCAE, v.5.0).^20^

### Noncompartmental PK analysis

Noncompartmental PK analysis (NCA) was performed to determine the plasma PK parameters of clofazimine, including the area under the time versus concentration curve (total exposure, AUC_0-24h_), peak concentration (*C*_max_), time to *C*_max_ (*T*_max_) and the (pre-dose) trough concentration (*C*_trough_), after the last loading dose of 300 mg.

### Comparator study without a loading dose regimen

We used PK data from a completed randomized clinical trial (PERC trial), executed at the Radboudumc, that compared the efficacy of a rifampicin-based regimen and a clofazimine-based regimen for MAC-PD.^21^ Participants in the clofazimine-arm of that study received a dose of 100 mg once daily (without loading dose) and PK samples were collected, at 2 and 6 h post-dose, after 1 and 4 months of treatment, similar to PK sampling in our study. Plasma concentrations of clofazimine in this PERC trial were measured with the same assay for clofazimine. We used the PK data from the PERC trial to compare the highest measured clofazimine concentrations with our study and to develop a population PK model.

### PK model development

Three previously developed population PK models were evaluated to describe the clofazimine PK.^18,22,23^ The model evaluation is described in more detail in Text S1. The model fit was not satisfactory and therefore, non-linear mixed-effects modelling was used to develop a pharmacokinetic model for clofazimine, based on the data from the current study and the PERC trial.^21^ Model development is described in Text S2.

### Simulations

The developed PK model was used to simulate alternative clofazimine loading dose regimens to shorten the time to steady-state-like concentrations. A virtual patient population of 1000 patients was generated for each simulated loading dose regimen. Body weight in the virtual patient population followed a log-normal distribution based on the combined demographics of our study and the PERC trial.^21^ Loading doses of 200, 300 and 400 mg once daily were assessed for periods of 4, 6 and 8 weeks in total, followed by a maintenance dose of 100 mg once daily. A regimen without a loading dose (100 mg once daily) served as the reference. We evaluated the time required to reach target concentrations for each of the dosing regimens. The selected target was 80% of the individual PK model predicted steady-state trough (pre-dose) concentrations, achieved with a dose of 100 mg once daily. We assumed that at 80% of steady-state concentrations no clinically relevant difference in effect could be expected compared to true steady-state. Moreover, with this target we aimed to prevent reaching peak concentrations (associated with QTc prolongation^24^) during the loading phase that exceeded steady-state peak concentrations, achieved with a dose of 100 mg once daily.

### Software

NCA was performed using Phoenix WinNonlin v.6.4 (Certara USA Inc., Princeton, NJ, USA). Descriptive statistics were performed using IBM SPSS Statistics for Windows (v.29.0). PK model development was performed using NONMEM v.7.5, and data management and post-processing of results were performed in R v.4.1.3.^25,26^ Perl-speaks NONMEM was used for visual predictive checks (VPC) generation, SCM analysis and the SIR procedure.

## Results

### Study population

A total of 12 participants were screened and all were enrolled in the study. Their demographics and baseline characteristics are depicted in Table 1. Four participants dropped out of the study. All dropouts occurred in period 2 of the study and none were deemed related to clofazimine (details are presented in Table S3). Adherence to clofazimine was high and ranged from 95% to 100%.

**Table 1.** Demographic and baseline characteristics

*N*	12
Age, years, median (range)	69 (39–82)
Body weight, kg, median (range)	62 (51–105)
Fat-free mass, kg, median (range)^a^	50 (38–70)
Male sex, *n* (%)	6 (50)
Race, *n* (%)	
Caucasian	10 (83)
Middle Eastern	2 (17)
Pulmonary NTM disease, *n* (%)	9 (75)
Extrapulmonary NTM disease, *n* (%)	3 (25)
Causative species (*n*)	
*M. avium* complex	7
*M. avium* complex and *M. abscessus*	1
*M. abscessus*	1
*M. chelonae*	1
*M. malmoense*	1
*M. xenopi*	1
Other antimycobacterial drugs used (*n*)	
Amikacin (intravenous)	8
Amikacin liposomal (inhalation)	1
Azithromycin	11
Clarithromycin	1
Ethambutol	9
Imipenem/cilastatin	2
Moxifloxacin	1
Rifampicin	7

^a^Calculated according to Janmahasatian *et al*.^27^

### NCA and descriptive PK

The geometric mean (GM) *C*_max_, *C*_trough_ and AUC_0-24h_ of clofazimine after the last loading dose of 300 mg were 0.87 mg/L, 0.50 mg/L and 16.1 mg h/L (Table 2), respectively. The GM highest measured concentrations after ∼1 (first dose of 100 mg) and 4 months of treatment were 0.75 and 0.93 mg/L. In the PERC trial, GM highest measured concentrations after 1 and 4 months were 0.39 and 0.78 mg/L, respectively (Table 2).

**Table 2.** Pharmacokinetic parameters of clofazimine in this study (C-LOAD) and the comparator trial (PERC)^21^

	Day 28 (± 2)	1 month^c^		4 months^d^	
	C-LOAD	C-LOAD	PERC	C-LOAD	PERC
	300 mg	100 mg	100 mg	100 mg	100 mg
PK parameter^a^	*n* = 12	*n* = 12	*n* = 19	*n* = 8	*n* = 16
*C*_max_ (mg/L)	0.87 (0.69–1.11)				
*T*_max_ (h)	6.0 (2.0–8.1)				
*C*_highest_ (mg/L)^b^	NA	0.75 (0.57–0.97)	0.39 (0.30–0.50)	0.93 (0.71–1.2)	0.78 (0.60–1.02)
*C*_trough_ (mg/L)	0.50 (0.39–0.64)				
AUC_0-24h_ (mg h/L)	16.1 (12.8–20.4)				

^a^PK parameters are depicted as GM (95% CI), except for *T*_max_ that is depicted as median (range).

^b^Due to the limited number of samples (2 and 6 h post-dose), the *C*_max_ may not have been captured. The highest measured concentration (*C*_highest_) was reported as an alternative PK parameter.

^c^C-LOAD, Day 28 visit + 1 day; PERC, 1 month ± 1 week (1 participant + 11 days).

^d^C-LOAD, 4 months ± 1 week; PERC, 4 months ± 2 weeks (1 participant − 16 days).

### Adverse events

A total of 151 AEs were reported in 12 participants, including 109 grade 1, 25 grade 2 and 17 grade ≥3 events (see Table 3). Clofazimine was well-tolerated and was not discontinued in any of the participants. The most common AEs were gastro-intestinal (GI; *n* = 28 in 11 participants), skin-related (*n* = 20 in 10 participants) and ototoxicity (*n* = 12 in eight participants). Seven serious AEs (SAEs) were reported in six participants. Five SAEs were unscheduled or prolonged hospitalizations and two SAEs were deaths. None of the SAEs were deemed related to the use of clofazimine (details are described in Table S4).

**Table 3.** Overview of AEs

	Period 1 (*n*)	Period 2 (*n*)
Total number of AEs	71	80
CTCAE severity grade^a^		
Grade 1	49	60
Grade 2	15	10
Grade ≥3	7	10
AEs contributing to >5% of total number of AEs		
Gastro-intestinal	15	13
Ototoxicity	4	8
Skin-related	9	11
Elektrolyte imbalance	1	9
QTc prolongation	6	3
Hypoalbuminaemia	6	2
Other	12	19
Total number of SAEs	2	5
Unscheduled hospitalization	1	3
Prolonged hospitalization	1	
Death		2
Related to clofazimine	0	0

^a^In case the severity of an AE changed over time (e.g. from grade 2 to 3), this was described as one AE with the highest reported severity grade. The start date of an AE was the first reported date of this AE.

### PK model development

In total, 112 clofazimine concentrations from our study (12 patients) and 67 concentrations (19 patients) from PERC were available for the development of the population PK model.

Clofazimine PK were best described by a two-compartment disposition model. Absorption was characterized by a depot and one transit compartment with first-order absorption into the central clofazimine compartment. Estimation of a separate absorption-rate constant and mean-transit time did not significantly improve the model fit. A combined proportional and additive error model described the residual error in the data best. Allometric scaling was included based on total body weight. A schematic representation of the PK model is presented in Figure S1.

**Figure 2.**
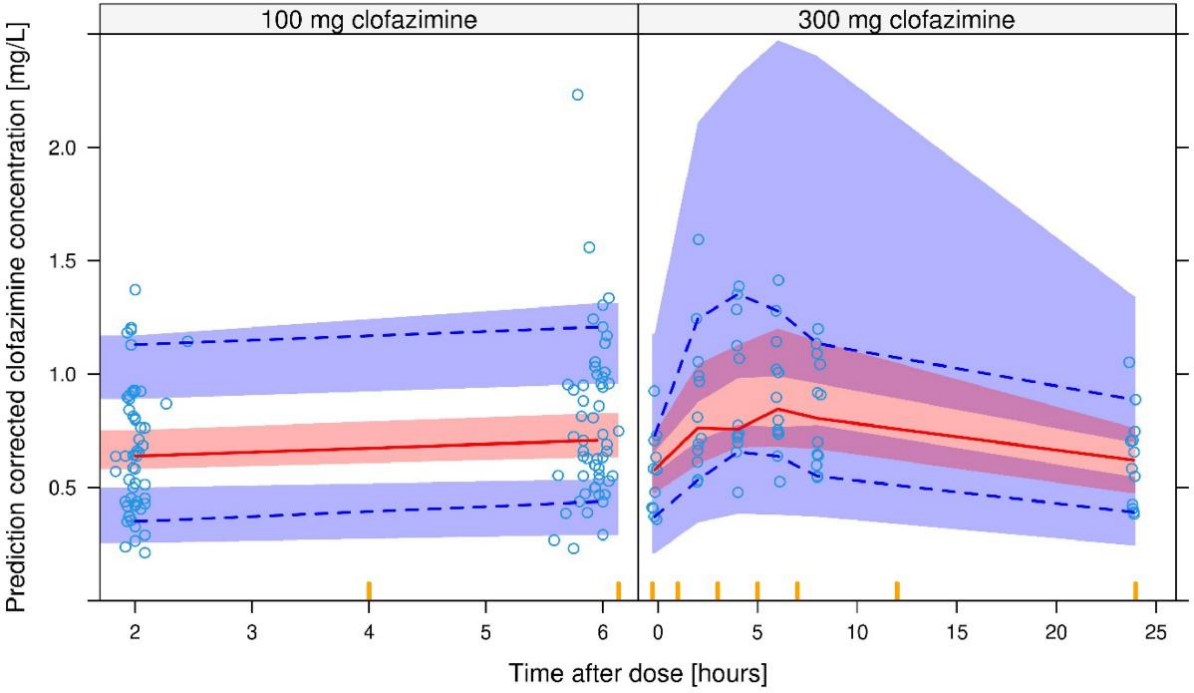
VPCs showing the observed fifth, 50th and 95th percentiles (lower dashed, solid middle and upper dashed lines, respectively) and the 95% CI for the same percentiles (shaded areas) calculated from simulated data using the final PK model. The left shows observations and model predictions after 100 mg dosing. The right shows observations and model predictions after 300 mg dosing. This figure appears in colour in the online version of *JAC* and in black and white in the print version of *JAC*.

Covariate analysis revealed that the bioavailability of clofazimine was ∼26% lower (95% CI: 9.2–40%) for 300 mg doses compared to 100 mg doses (*P* < 0.01). No other tested covariate significantly affected the PK of clofazimine (other tested covariates were: rifampicin co-administration, age, sex and pulmonary versus extrapulmonary disease). Moreover, no statistically significant differences in key clofazimine PK parameters (absorption rate, volume of distribution, clearance and bioavailability) were detected between our study and the PERC trial. Final PK model parameters are presented in Table 4, and VPC are presented in Figure 2. Relatively large confidence intervals were observed around the fifth, 50th and 95th percentiles for a clofazimine dose of 300 mg (Figure 2, right), due to a limited number of observations. Better performance was observed for a dose of 100 mg, representing more observations from both C-LOAD and PERC (Figure 2, left). In addition, the model described the accumulation of clofazimine over time well (Figure S3). Overall, the model described the data well. Goodness-of-fit plots and final model code are presented in Figure S2 and Text S3, respectively. Model-based estimated exposure parameters of clofazimine are depicted in Table S5.

**Table 4.** Final pharmacokinetic model parameters for clofazimine

Parameter	Typical value (95% CI)^a^
Fixed effects^b^	
Clearance (L/h)	4.17 (2.81–5.79)
Central volume of distribution (L)	460 (370–608)
Absorption-rate constant (1/h)	0.683 (0.502–0.940)
Intercompartmental clearance (L/h)	24.6 (18.1–32.9)
Peripheral volume of distribution (L)	10100 (7670–13000)
Effect of high-dose on bioavailability (%)	73.6 (59.6–91.8)
Random effects^c^	
Inter-individual variability in clearance (CV%)	85.6 (55.9–143)
Inter-individual variability in absorption-rate constant (CV%)	80.6 (47.8–139)
Inter-occasion variability in bioavailability (CV%)	79.3 (59.7–122)
Residual error	
Proportional error (%)	8.00 (5.95–10.5)
Additive error (mg/L)	0.00174 (0.000701–0.00278)

^a^95% confidence interval obtained using the SIR method.

^b^All disposition parameters are reported as apparent disposition parameters. Volumes and clearances are allometrically scaled using fixed exponents of 1 and 0.75, respectively, with 70 kg as reference total body weight.

^c^Interindividual variability CV% calculated as √(exp(OM^2^) − 1).

### Simulations

**Figure 3.**
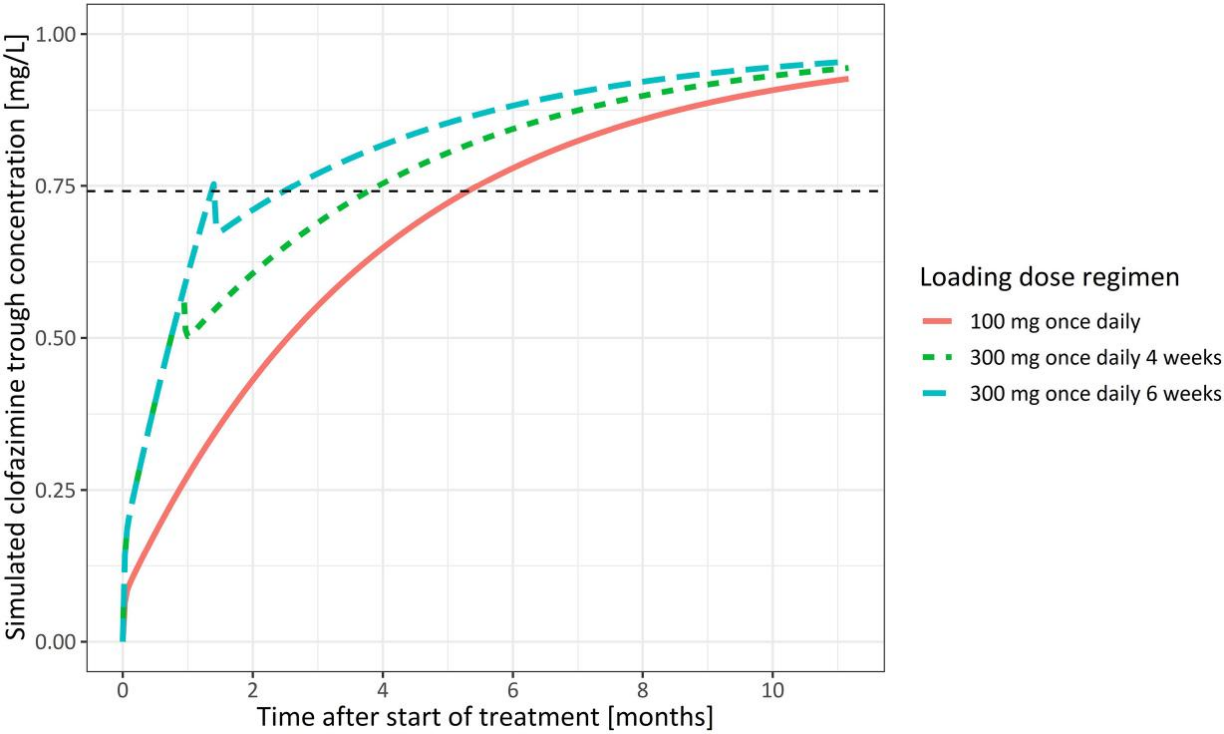
Simulated clofazimine trough (pre-dose) concentration versus time curves for a typical patient (65 kg) following three different dosing regimens; 100 mg once daily (reference), 300 mg once daily for 4 weeks followed by 100 mg once daily and 300 mg once daily for 6 weeks followed by 100 mg once daily. The dashed line represents the target concentration, i.e. 80% of the predicted steady-state trough concentration. This figure appears in colour in the online version of *JAC* and in black and white in the print version of *JAC*.

In our virtual population of 1000 patients, the median time to reach the target concentration (80% of individual steady-state concentration) for clofazimine (100 mg once daily) without a loading dose was 5.3 months. When using the loading dose regimen investigated in this study, 300 mg once daily for 4 weeks, the median time to reach the target concentration was reduced to 3.8 months. Extending the loading phase to 6 weeks decreased the median time to target concentration to 1.4 months. No relevant additional gain in time to target concentration was predicted with either a longer loading phase of 8 weeks or a higher loading dose of 400 mg. Peak concentrations during the loading phase for loading regimens up to 6 weeks were similar compared to peak concentrations at steady-state with a dose of 100 mg once daily. The results of the simulated dosing regimens are depicted in Table 5. To illustrate the effects of different loading dose regimens, concentration-time profiles for a typical patient of 65 kg are presented in Figure 3.

**Table 5.** Results for the simulated loading dose regimens of clofazimine

Dose regimen^a^	Time to target concentration^b^ (months)	Highest *C*_max_ during loading dose period (mg/L)	*C*_max_ during steady-state (mg/L)
100 mg once daily (reference)	5.3 (0.93–8.1)	NA	1.1 (0.29–2.7)
200 mg once daily, 4 weeks	4.6 (0.53–7.9)	0.75 (0.42–1.6)	1.1 (0.32–2.9)
300 mg once daily, 4 weeks	3.8 (0.33–7.6)	0.96 (0.55–2.1)	1.1 (0.34–2.8)
400 mg once daily, 4 weeks	3.5 (0.20–7.6)	1.1 (0.55–2.3)	1.1 (0.28–2.8)
200 mg once daily, 6 weeks	3.9 (0.50–7.6)	0.87 (0.43–1.9)	1.1 (0.29–2.8)
300 mg once daily, 6 weeks	1.4 (0.30–7.3)	1.1 (0.58–2.3)	1.1 (0.32–2.7)
400 mg once daily, 6 weeks	1.2 (0.23–7.3)	1.2 (0.63–2.5)	1.1 (0.30–2.9)
200 mg once daily, 8 weeks	1.9 (0.53–7.4)	1.0 (0.47–2.0)	1.1 (0.30–2.8)
300 mg once daily, 8 weeks	1.4 (0.30–7.0)	1.3 (0.64–2.6)	1.1 (0.31–2.9)
400 mg once daily, 8 weeks	1.3 (0.23–7.0)	1.4 (0.70–2.9)	1.1 (0.29–3.1)

^a^All loading dose regimens were followed by a maintenance dose of 100 mg once daily.

^b^Target concentration: 80% of the individual PK model predicted steady-state trough (pre-dose) concentrations, achieved with a dose of 100 mg once daily.

All results are depicted as median (2.5th–97.5th percentiles).

NA, not applicable.

## Discussion

A clofazimine dose of 300 mg once daily for 4 weeks led to faster attainment of steady-state-like concentrations, as compared to no loading dose regimen, and was safe and well-tolerated. The PK model derived from our data revealed that a 300 mg once daily dose for 6 weeks is expected to further decrease the time to steady-state-like concentrations. These results show the usefulness and feasibility of a loading dose of clofazimine, which could be implemented in patient care and evaluated in follow-up studies.

After the loading phase, a relatively small difference in the highest measured clofazimine concentrations between 1 and 4 months of treatment (0.75 versus 0.93 mg/L) was observed in this study with a dose of 100 mg. By contrast, a larger (2-fold) difference of the highest measured clofazimine concentrations between 1 and 4 months of treatment (0.39 versus 0.78 mg/L) was observed in the PERC trial with a clofazimine dose of 100 mg once daily (without loading phase).^21^ These results illustrate that a loading dose yields steady-state-like concentrations faster.

AEs were reported frequently throughout the study, which is common among patients with NTM diseases undergoing treatment,^28^ but none resulted in discontinuation of clofazimine. Although attributing AEs to a specific antibiotic in multidrug regimens is challenging, AEs associated with clofazimine include QTc prolongation, gastro-intestinal (GI) and skin-related effects.^6^ QTc prolongation was observed in nine participants, including two grade 3 events. All patients used two antimycobacterial drugs that can cause QTc prolongation (clofazimine and a macrolide or moxifloxacin), and in some cases additional QTc-prolongating drugs. Our observations are in line with previous reports, especially when clofazimine is combined with other QTc-prolongating drugs.^29^ GI AEs frequently occurred, similar to previous reports on clofazimine-based regimens.^30^ They were mostly mild, with the exception of six grade 3 events (in three participants). Skin-related AEs characteristic for clofazimine (discoloration, dryness and pruritis), were also common but generally mild. Overall, no unexpected events related to clofazimine were reported in this study. Of course, the number of patients receiving a loading dose in our study was small for the purpose of a safety evaluation. However, in the treatment of leprosy and DR-TB there is experience with a daily clofazimine dose of 300 mg (up to 600 mg for leprosy) for months and even years.^14–17^ Furthermore, steady-state concentrations were not exceeded during the loading phase, which further supports the safety of the loading dose regimen.

The population PK model for clofazimine in NTM patients showed that clofazimine PK were characterized by a large volume of distribution (∼10 000 L) and a long terminal half-life (∼80 days), consistent with previous reports.^6,18^ An important finding was that the bioavailability of clofazimine was 26% lower with a dose of 300 mg compared to a 100 mg dose. This could be explained by dose-dependent saturation of absorption, rather than a previously suggested time-dependent effect,^18^ and is supported by previous observations in a study on faecal excretion in relation to different doses of clofazimine.^31^ This finding indicates there are limits to the dose that can be administered and further dose increases (above 300 mg once daily) may not lead to substantially higher exposure in plasma.

Our loading dose simulations demonstrate that a large improvement can be achieved in the time required to reach steady-state-like concentrations when using a loading dose regimen. The loading dose investigated in this study reduced the time to target concentrations by 1.5 months, as compared to not using a loading dose (100 mg once daily). Importantly, the peak concentration (associated with QTc prolongation^24^) during the loading phase did not surpass the peak concentrations at steady-state, reaffirming the safety of the loading dose regimen. The optimal loading dose regimen was determined to be a dose of 300 mg once daily for 6 weeks. This regimen reduces the time to target concentrations by 3.9 months compared to not using a loading dose, without exceeding steady-state peak concentrations. A higher loading dose of 400 mg once daily did not further improve the time to target concentrations in our simulations due to a further decrease in bioavailability. It should be noted that this simulation is less certain as it builds on extrapolation outside of the investigated dose range. Furthermore, multiple daily dosing (e.g. 200 mg twice daily) may circumvent bioavailability limitations. We deemed once daily dosing preferable in the context of multidrug regimens and therefore we did not assess multiple daily dosing.

Previously, a different loading dose regimen of 200 mg once daily for 2–4 weeks has been proposed for the treatment of TB based on model simulations.^18^ This loading dose regimen was based on a target concentration of 0.25 mg/L. This target was derived from a murine TB model in which sustained antimicrobial activity after treatment cessation was associated with concentrations of ≥0.25 mg/L.^32^ However, there is no evidence that this target is associated with maximum effect against *Mycobacterium tuberculosis*. In general, there are limited data on exposure–response relationships for clofazimine in TB. In patients with MDR-TB an AUC_0-24h_/MIC ratio of ≥50 was associated with faster culture conversion.^33^ In another study, patients in whom culture conversion was achieved had higher AUC_0-24h_/MIC ratios after 2 and 6 months of treatment (AUC_0-24h_/MIC: 116 versus 64 and 101 versus 52, respectively).^34^ For NTM disease, data on exposure–response relationships are even more scarce. In two studies in NTM-PD, lower MICs (≤0.25 mg/L) were associated with better outcomes.^35,36^ In the PERC trial, patients in whom culture conversion was achieved, had higher peak clofazimine concentrations (0.95 versus 0.51 mg/L).^21^ While these data suggest that higher clofazimine exposures may be favourable, the optimal exposure–response relationships (and thus exposure targets) of clofazimine remain to be established. As clofazimine-based treatment regimens with a standard dose of 100 mg once daily have shown promising outcomes in NTM disease,^11–13,21,30,37^ we deemed steady-state concentrations that are achieved with this dose as the most suitable target for our loading dose strategy. However, we recognize that the current standard dose of 100 mg once daily is not well substantiated.^14^ The evaluation of exposure-response relationships of clofazimine should be a goal of future studies to define exposure targets and to further establish optimal dosing of clofazimine for NTM disease.

Our study had limitations. The sample size was small and the study had no control arm without a loading dose. However, we believe that the PERC trial served very well as a comparator study because it was performed in the same setting, had a similar patient population (except three patients with extrapulmonary NTM disease in our study) and clofazimine plasma concentrations had been measured with the same analytical method.^21^ The appropriate choice of the PERC trial as comparator was substantiated by the absence of differences in PK parameters between our study and PERC. A notable difference from the PERC trial is that seven patients in our study used rifampicin, five of whom used it close to the PK sampling days. One study reported a limited reduction in bioavailability of clofazimine (22%) when combined with rifampicin,^23^ whereas other studies suggest that rifampicin has no effect on the exposure to clofazimine.^38,39^ In our study, rifampicin use was not identified as a significant predictor of clofazimine exposure, but the sample size was small.

There is significant variability in reported PK of clofazimine between different studies and patient populations.^18,23,40^ Moreover, existing population PK models did not describe our data well (Text S1). As a result, it is unknown how our specific recommendations on the optimal loading dose regimen translate to other populations. However, we believe that our finding on the usefulness and feasibility of a clofazimine loading dose strategy is universally applicable.

In conclusion, a 4-week loading dose regimen of 300 mg once daily significantly reduced the time to steady-state-like concentrations of clofazimine and was safe and well-tolerated. Extending the loading phase to 6 weeks is expected to yield additional time benefit. These results show the feasibility of a loading dose of clofazimine, which could be implemented in patient care and evaluated in follow-up studies.

## Supplementary Material

dkae309_Supplementary_Data

## Supplementary data

Figures S1 to S3, Tables S1 to S5 and Text S1 to S3 are available as Supplementary data at *JAC* Online.
