# Supplementary material for: A loading dose of clofazimine to rapidly achieve steady-state-like concentrations in patients with nontuberculous mycobacterial disease
Source: J Antimicrob Chemother. 2024 Oct 8;79(12):3100–8. doi: 10.1093/jac/dkae309 (PMC11638672; doi:10.1093/jac/dkae309)
Supplement: dkae309_Supplementary_Data [file dkae309_supplementary_data.docx]

**Supplementary material**

Overview of content:

Methods:

Table S1. Overview of inclusion and exclusion criteria
Table S2. Validation parameters of the bioanalytical assay for clofazimine

Text S1. Evaluation of previously published population PK models for clofazimine
Text S2. Development of pharmacokinetic model

Results:

Table S3. Description of dropouts
Table S4. Description of SAEs
Figure S1. Schematic representation of the clofazimine pharmacokinetic model
Table S5: Population PK model derived clofazimine PK parameters at month 1 and 4 of clofazimine treatment for the C-LOAD and PERC study.
Figure S2: Goodness-of-fit plots for the final clofazimine population PK model
Figure S3: Visual Predictive Checks (VPCs) of clofazimine accumulation over time on treatment
Text S3: Model code

**Methods**

| **Table S1. Overview of inclusion and exclusion criteria** |
| --- |
| **Inclusion criteria:** |
| The participant is diagnosed with pulmonary or extrapulmonary NTM disease and is eligible for treatment with clofazimine |
| The participant is at least 18 years of age |
| The participant has a body weight (in light clothing and with no shoes) of at least 45 kg |
| The participant is able and willing to provide written, informed consent |
| **Exclusion criteria:** |
| The participant is in poor general condition where participation in the study cannot be accepted per discretion of the Investigator |
| There is evidence showing the participant has clinically significant metabolic, gastrointestinal, or other abnormalities that could possibly alter the PK of clofazimine |
| The participant is diagnosed with cystic fibrosis |
| The participant has a prolongation of the QTc interval, > 450 milliseconds for males and > 460 milliseconds for females, on the screening ECG |
| The participant has abnormal alanine aminotransferase (ALT) and/or aspartate transferase (AST) levels of > 3 times the upper limit of the laboratory reference range at screening |
| The participant is pregnant or is using inadequate contraceptive measures (if applicable) |
| The participant is breastfeeding (if applicable) |
| The participant has a known or suspected, current drug or alcohol abuse, that is, in the opinion of the Investigator, sufficient to compromise the safety or cooperation of the patient |
| The participant has as history of allergy/hypersensitivity to clofazimine |
| The participant has received clofazimine in the past 3 months before inclusion with the exception of short-term use of no more than 7 days in the period of 1 to 3 months before inclusion |

| **Table S2. Validation parameters of the bioanalytical assay for clofazimine** | | | | | |
| --- | --- | --- | --- | --- | --- |
| Compound | Calibration range | Intraday | | Interday | |
|  | Concentration  (mg/L) | Accuracy  % | Imprecision  % | Accuracy  % | Imprecision  % |
| Clofazimine | 0.05 - 10.0 | 95.1-104.6 | 3.0-6.9 | 98.9-102.3 | 0.9-3.2 |

**Text S1. Evaluation of previously published population PK models for clofazimine**

Literature search identified three population pharmacokinetic models for clofazimine.

In summary, Abdelwahab *et al.*^1^ pooled data from two studies (PROBeX and a phase IIa clinical trial ^2, 3^) to study the clofazimine PK in participants with pulmonary tuberculosis. In total, 1570 observations from 139 participants were included, from both sparse and intensive PK sampling. A three-compartmental PK model with an estimated number of transit compartments was reported. Allometric scaling was based on total body weight for clearances, and one peripheral volume, on fat mass for the second peripheral volume, and on fat-free mass for the central volume, standardized to their respective medians. Furthermore, an auto-inhibitory effect of clofazimine on P-glycoprotein was hypothesized, leading to a decreased bioavailability during the first days on treatment.

Faraj et al.^4^ used data from a phase IIa clinical trial in participants with pulmonary tuberculosis.^3^ In total, data 14 participants were included with intensive PK sampling. The authors reported a two-compartmental PK model with a lag time absorption model and allometric scaling of clearance and volume parameters using total body weight standardized to the population median, and fixed exponents of 0.75 and 1, respectively.

Watanabe *et al.*^5^ used data form a trial in participants with a pulmonary NTM infection. In total, 144 PK observations from 45 participants were included for model development. All PK observations were pre-dose samples. The authors reported a one-compartmental PK model with allometric scaling of the clearance using an estimated exponent of 1.16 based on total bodyweight and of the volumes using an estimated exponent of 1.55 based on body mass index, both standardized to the population median. Furthermore, rifampicin co-administration was estimated to reduce the bioavailability of clofazimine by 22%.

To evaluate the ability of the models to describe the data from this study and the PERC study, a two-step approach was used. First, an evaluation of the PK model was performed using the reported pharmacokinetic parameter estimates. Secondly, key pharmacokinetic parameters (clearance, volume of distribution, absorption, bioavailability, and their respective variability) were re-estimated for each model. For both steps, VPCs were created to assess the model fit. The VPCs for each model before and after the estimation of the key pharmacokinetic parameters are shown below. All VPC show the observed 5^th^, 50^th^, and 95^th^ percentiles (lines) and the 95% CI for the same percentiles (shaded areas) calculated from simulated data using the respective PK model.

**Abdelwahab *et al.***

**No estimation Key parameters re-estimated**


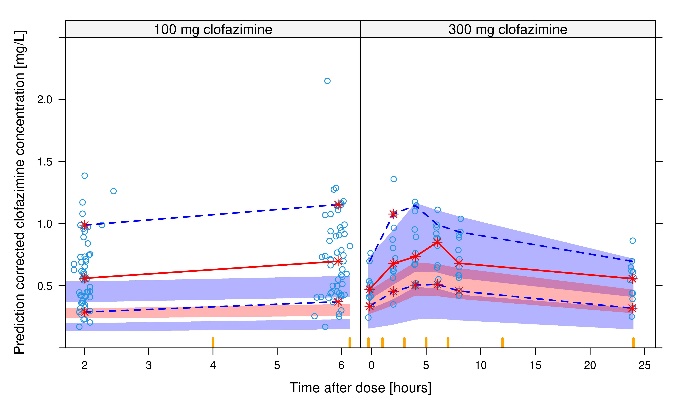

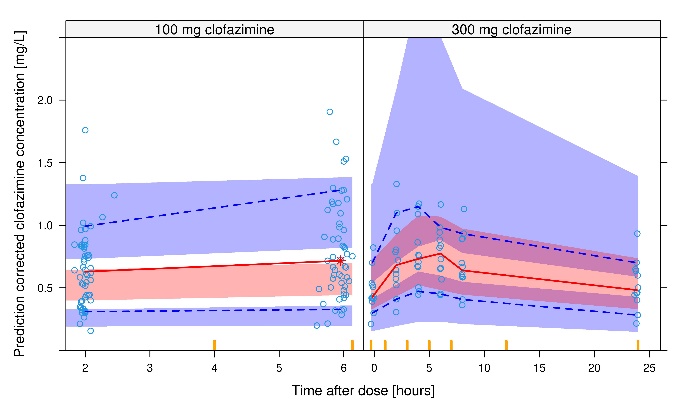


**Faraj *et al.***

**No estimation Key parameters re-estimated**


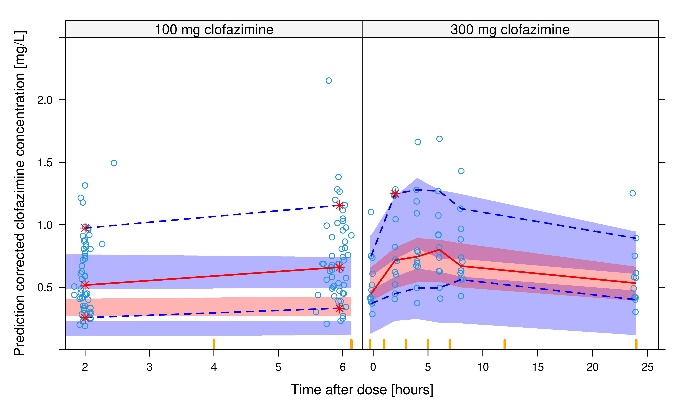

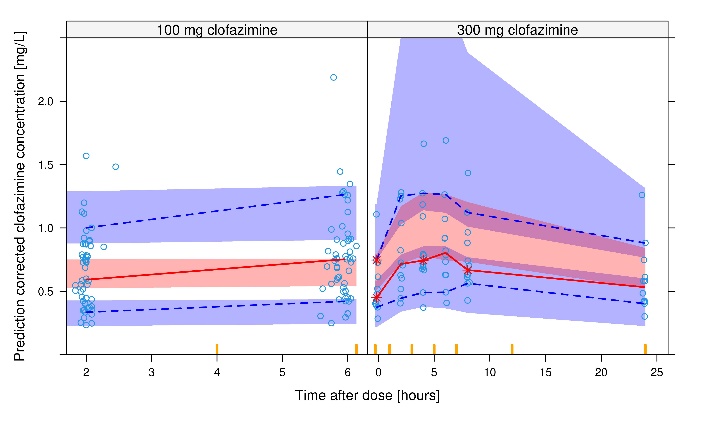


**Watanabe *et al.***

**No estimation Key parameters re-estimated**


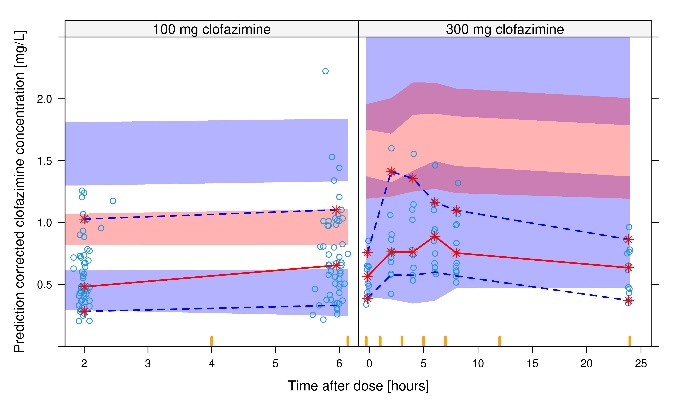

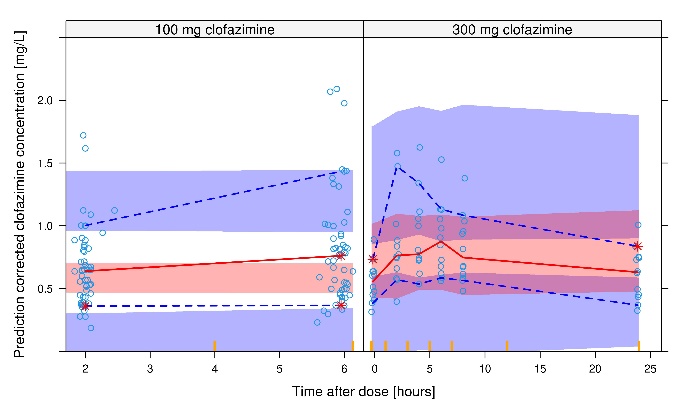


The population PK models of Abdelwahab *et al.* and Faraj *et al.* both showed an underprediction of the clofazimine concentration before re-estimation. Conversely, Watanabe *et al.* overpredicted the clofazimine concentrations before re-estimation. Re-estimation of the PK modes showed that the models from Abdelwahab *et al.* and Faraj *et al.* were able to describe the shape of the concentration-time profile well, but were not able to describe both the 100 mg once daily dose and the 300 mg once daily dose. The model from Watanabe *et al.* did not follow the general shape of the clofazimine concentration-time profile well. Concluding, none of the evaluated models were able to accurately describe the clofazimine PK in this study combined with the PERC study.

**Text S2. Development of pharmacokinetic model**

One-, two-, and three-compartmental disposition models were investigated. First-order, zero-order, and transit compartment models were tested for their ability to describe the clofazimine absorption. First-order clearance was assumed to take place from the central clofazimine compartment. Interindividual variability was assumed to be log-normally distributed.

Allometric scaling was implemented using fixed standard exponentials of 1 for volumes and 0.75 for clearances. Total body weight, fat-free mass,^6^ and fat mass were investigated as body size descriptors for use in allometric scaling. Analysis of other predictors of the PK of clofazimine (covariates) were assessed using stepwise covariate modeling (SCM). A forward significance criterion of p<0.05 and a backward criterion of p<0.01 were used as significance cutoffs. Explorative covariates were: dose of clofazimine, rifampicin co-administration, age, sex, and pulmonary versus extrapulmonary disease.

The 95% confidence interval for all model parameters was determined using the sampling importance resampling (SIR) procedure.^7^ Model evaluation was performed using goodness-of-fit plots as well as visual predictive checks (VPCs). Additional VPCs of the accumulation of clofazimine over time on treatment are depicted in figure S3. A decrease in objective function value of < -3.84 was considered significant at the p=0.05 level.

**Results**

| **Table S3. Description of dropouts** | |
| --- | --- |
| 1 | One participant died due to a massive pulmonary hemorrhage |
| 2 | One participant died due to respiratory insufficiency caused by an acute COPD exacerbation |
| 3 | One participant withdrew because of clinical deterioration leading to cessation of antimycobacterial treatment |
| 4 | One participant withdrew at their own request |

| **Table S4. Description of SAEs** | |
| --- | --- |
| 1 | Hospital admission because of clinical deterioration (weight loss, dyspnea) |
| 2 | Death due to a massive pulmonary hemorrhage |
| 3 | Hospital admission because of clinical deterioration (weight loss, dyspnea and fever) |
| 4 | Hospital admission due to nausea (and vomiting), most likely related to the use of imipenem/cilastatin |
| 5 | Acute exacerbation of COPD resulting in prolonged hospitalization and shortly thereafter death |
| 6 | Hospital admission due to influenza A infection |
| 7 | Prolonged hospitalization due to tubulo-interstitial nephritis, related to the use of ethambutol |

| **Table S5: Population PK model derived clofazimine PK parameters at month 1 and 4 of clofazimine treatment for this study (C-LOAD) and the comparator PERC study.^8^** | | | | | |
| --- | --- | --- | --- | --- | --- |
|  | Day 28 (± 2) | 1 month^b^ | | 4 months^c^ | |
|  | C-LOAD | C-LOAD | PERC | C-LOAD | PERC |
|  | 300mg | 100 mg | 100 mg | 100 mg | 100 mg |
| PK parameter ^a^ | n=12 | n = 12 | n = 19 | n = 8 | n = 16 |
| AUC_0-24h_ (mg*h/L) | 16.0  (12.6-20.3) | 14.6  (11.5-18.5) | 7.75  (6.18-9.72) | 19.8  (15.1-25.9) | 15.8  (12.4-20.0) |
| C_max_ (mg/L) | 0.832  (0.649-1.07) | 0.734  (0.575-0.937) | 0.399  (0.317-0.502) | 0.930  (0.720-1.20) | 0.767  (0.606-0.971) |
| T_max_ (h) | 4.28  (3.37-5.44) | 3.93  (3.22-4.80) | 4.99  (4.26-5.83) | 3.76  (2.83-5.01) | 5.26  (4.68-5.92) |
| C_trough_ (mg/L) | 0.507  (0.406-0.633) | 0.517  (0.409-0.654) | 0.233  (0.195-0.280) | 0.716  (0.522-0.982) | 0.499  (0.393 -0.635) |

^a^ PK parameters are depicted as geometric mean (95% CI)
^b^ C-LOAD, Day 28 visit + 1 day; PERC, 1 month ± 1 week (1 participant + 11 days)
^c^ C-LOAD, 4 months ± 1 week; PERC, 4 months ± 2 weeks (1 participant - 16 days)


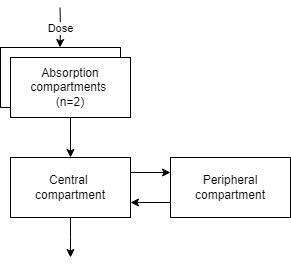


**Figure S1. S****chematic representation of the clofazimine (CFZ) pharmacokinetic model**


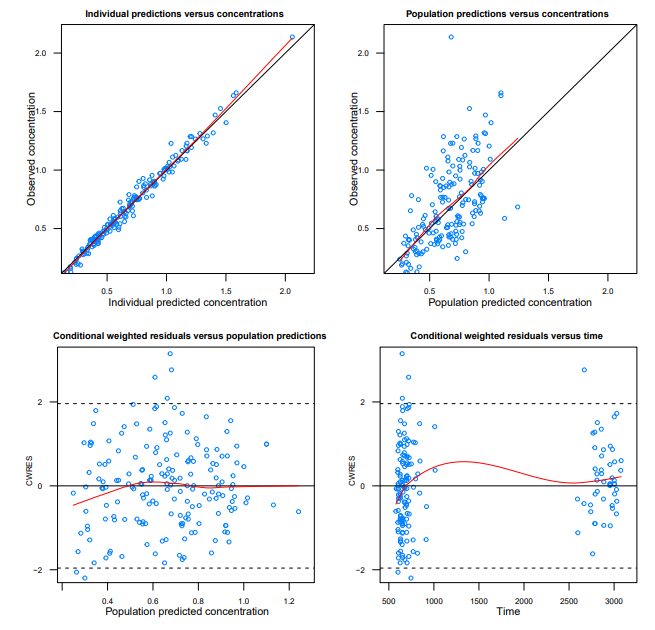


**Figure S2:** Goodness-of-fit plots for the final clofazimine population PK model. Top left: Observed clofazimine concentrations versus individual model predicted concentrations. Top right: Observed clofazimine concentrations versus population model predicted concentrations. Bottom left: Conditional weighted residuals versus population model predicted concentrations. Bottom right: Conditional weighted residuals over time after the start of treatment.


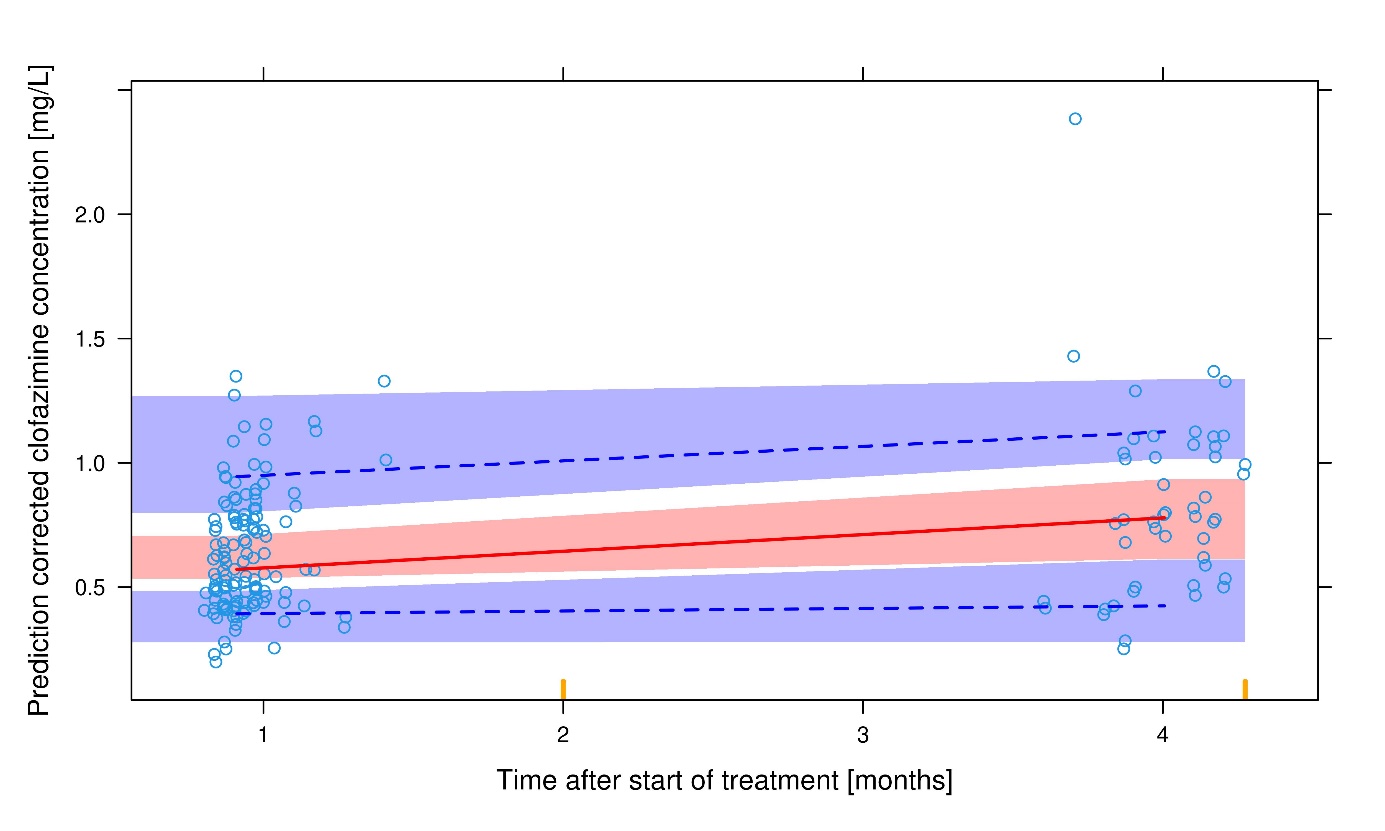


**Figure S3:** Visual Predictive Checks (VPCs) of clofazimine accumulation over time on treatment showing the observed 5^th^, 50^th^, and 95^th^ percentiles (lower dashed, solid middle, and upper dashed lines, respectively) and the 95% CI for the same percentiles (shaded areas) calculated from simulated data using the final PK model.

**Text S3: Model code**

$PROBLEM Clofazimine population pharmacokinetic model

$INPUT ID TIME DV EVID MDV AMT OCC WT STUDY

$DATA … .csv IGNORE=@

;Data dictionary

;ID Subject ID number

;TIME Time after start treatment (h)

;DV Observed CFZ concentration (mg/L)

;EVID Event ID

;MDV Missing dependent variable

;AMT Amount (mg)

;OCC Occasion (0 for no PK sample, 1/2/3 for each PK day)

;WT Weight (kg)

;STUDY Study identifier for C-LOAD =0, for PERC =1

$SUBROUTINES ADVAN5

$MODEL

COMP=(DEPOT)

COMP=(TRANSIT)

COMP=(CENTRAL)

COMP=(PERIPHERAL)

$PK

;Allometric scaling parameters

AlloCL = (WT/70)**0.75

AlloV = (WT/70)**1

;PK parameters

TVCL = THETA(1)

TVV = THETA(2)

TVKTR = THETA(3)

TVQ = THETA(4)

TVVp1 = THETA(5)

highdoseEFF=1

IF(highdose.EQ.1) highdoseEFF = THETA(6)

;Variability

IIVCL = EXP(ETA(1))

IIVKTR = EXP(ETA(2))

IOVF = 1

IF(OCC.EQ.1)IOVF = EXP(ETA(3))

IF(OCC.EQ.2)IOVF = EXP(ETA(4))

IF(OCC.EQ.3)IOVF = EXP(ETA(5))

; Parameters

CL = TVCL * IIVCL * AlloCL

V = TVV * AlloV

KTR = TVKTR * IIVKTR

Q = TVQ * AlloCL

Vp1 = TVVp1 * AlloV

;Bioavailability

F1 = 1* IOVF *highdoseEFF

;Rate constants

K12 =KTR

K23 =KTR

K30 =CL/V

K34 =Q/V

K43 =Q/Vp1

$ERROR

IPRED = A(3)/V

;Error model

PROPERR= EPS(1)

ADDERR= EPS(2)

Y = IPRED *(1+ PROPERR)+ADDERR

$THETA

(0, 4.17) ; 1 CL

(0, 460) ; 2 V

(0, 0.683) ; 3 KA

(0, 24.6) ; 4 Q

(0, 10100) ; 5 Vp1

(0, 0.736) ;6 F highdose

$OMEGA

0.55 ; 1 IIV CL

0.501 ; 2 IIV KTR

$OMEGA BLOCK(1)

0.488 ; 3 IOV F

$OMEGA BLOCK(1) SAME

$OMEGA BLOCK(1) SAME

$SIGMA

0.00639 ; Proportional error

0.00174 ; Additive error

$ESTIMATION METHOD=1 INTER MAXEVAL=9999 NOABORT PRINT=1

$COVARIANCE

$TABLE …..

**References**

1. Abdelwahab MT, Wasserman S, Brust JCM *et al.* Clofazimine pharmacokinetics in patients with TB: dosing implications. *J Antimicrob Chemother* 2020; **75**: 3269-77.

2. Brust JCM, Gandhi NR, Wasserman S *et al.* Effectiveness and Cardiac Safety of Bedaquiline-Based Therapy for Drug-Resistant Tuberculosis: A Prospective Cohort Study. *Clin Infect Dis* 2021; **73**: 2083-92.

3. Diacon AH, Dawson R, von Groote-Bidlingmaier F *et al.* Bactericidal activity of pyrazinamide and clofazimine alone and in combinations with pretomanid and bedaquiline. *Am J Respir Crit Care Med* 2015; **191**: 943-53.

4. Faraj A, Svensson RJ, Diacon AH, Simonsson USH. Drug Effect of Clofazimine on Persisters Explains an Unexpected Increase in Bacterial Load in Patients. *Antimicrob Agents Chemother* 2020; **64**.

5. Watanabe F, Furuuchi K, Hanada K *et al.* Pharmacokinetics and Adverse Effects of Clofazimine in the Treatment of Pulmonary Non-Tuberculous Mycobacterial Infection. *Antimicrob Agents Chemother* 2022; **66**: e0044122.

6. Janmahasatian S, Duffull SB, Ash S *et al.* Quantification of lean bodyweight. *Clin Pharmacokinet* 2005; **44**: 1051-65.

7. Dosne AG, Bergstrand M, Harling K, Karlsson MO. Improving the estimation of parameter uncertainty distributions in nonlinear mixed effects models using sampling importance resampling. *J Pharmacokinet Pharmacodyn* 2016; **43**: 583-96.

8. Zweijpfenning SMH, Aarnoutse R, Boeree MJ *et al.* Safety and Efficacy of Clofazimine as an Alternative for Rifampicin in Mycobacterium avium Complex Pulmonary Disease Treatment: Outcomes of a Randomized Trial. *Chest* 2024; **165**: 1082-92.
